# [2.2]Paracyclophane derivatives containing tetrathiafulvalene moieties

**DOI:** 10.3762/bjoc.11.207

**Published:** 2015-10-15

**Authors:** Laura G Sarbu, Lucian G Bahrin, Peter G Jones, Lucian M Birsa, Henning Hopf

**Affiliations:** 1 Department of Chemistry, “Al. I. Cuza” University of Iasi, 11 Carol I Bv., RO-700506 Iasi, Romania; 2 Institute of Organic Chemistry, Technical University of Braunschweig, Hagenring 30, D-38106 Braunschweig, Germany; 3 Institute of Inorganic and Analytical Chemistry, Technical University of Braunschweig, Hagenring 30, D-38106 Braunschweig

**Keywords:** dithiocarbamates, 1,3-dithiolium salts, [2.2]paracyclophane, regioselective bromination, stereoisomers, tetrathiafulvalenes

## Abstract

The synthesis of [2.2]paracyclophane derivatives containing tetrathiafulvalene units has been accomplished by the coupling reaction of 4-([2.2]paracyclophan-4-yl)-1,3-dithiol-2-thione in the presence of trimethylphosphite. The 1,3-dithiol-2-thione derivative was in turn synthesized by the regioselective bromination of 4-acetyl[2.2]paracyclophane, then through the corresponding dithiocarbamates and 1,3-dithiolium salts.

## Introduction

Tetrathiafulvalene (TTF) and its derivatives have been extensively studied with respect to their applications as organic metals and superconductors [1–2]. These properties are a consequence of the π-donor properties of TTF and of its important intermolecular interactions in the solid state through extended π-orbitals. The design of new tetrathiafulvalene derivatives has targeted those systems where the intermolecular interactions between planar molecules are more effective and the solid-state architecture tends to be as stacks or layers with their long axes mutually parallel.

[2.2]Paracyclophane and its derivatives has been the subject of particular interest since their discovery, more than six decades ago [3–5]. Most of the unique properties of these cyclophanes are the result of the rigid framework and the short distance between the two aromatic rings within the [2.2]paracyclophane unit. Besides investigations of the geometry and of transannular interactions, special attention has been paid to the ability of these compounds to form charge-transfer complexes [6–9].

A recent report has appeared concerning the synthesis of a [2.2]paracyclophane doubly substituted by a dimeric tetrathiafulvalene in a *pseudo-ortho* substitution pattern [10]. This compound exhibited novel chiroptical properties. Prompted by these observations, we decided to investigate the synthesis of a tetrathiafulvalene that incorporates two [2.2]paracyclophane units.

## Results and Discussion

**Scheme 1:**
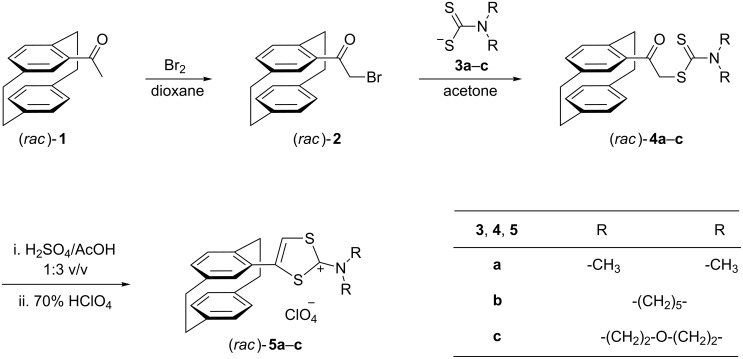
Synthesis of 2-*N*,*N*-dialkylamino-4-([2.2]paracyclophan-4-yl)-1,3-dithiol-2-ylium perchlorates **5**.

The synthesis of [2.2]paracyclophanes containing a tetrathiafulvalene moiety, follows a general route that involves the synthesis of the corresponding [2.2]paracyclophane-substituted 1,3-dithiol-2-ylium salts. These compounds are known to provide tetrathiafulvalenes under specific conditions [11–13]. The synthetic strategy for the incorporation of the 1,3-dithiol-2-ylium ring in the [2.2]paracyclophane framework involves a three-step reaction sequence, starting from 4-acetyl[2.2]paracyclophane (**1**) (Scheme 1).

The first step consists of the regioselective monobromination of 4-acetyl[2.2]paracyclophane (**1**) at the α-position to the carbonyl group to form 2-bromo-1-([2.2]paracyclophan-4-yl)ethan-1-one (**2**). The starting 4-acetyl[2.2]paracyclophane has been prepared according to the reported procedure [14]. In order to avoid the formation of side products during the bromination process [15], the synthesis of compound **2** has been accomplished using the molecular complex of bromine with dioxane [16]. However, although this reagent has often been employed in mild and selective bromination reactions [17], there are some difficulties concerning its isolation and handling [18]. In order to avoid these problems, we generated the reagent by mixing one equivalent of bromine with one equivalent of dioxane and adding dry dioxane to the resulting solid until the dissolution was complete. This solution was then added dropwise at room temperature to a solution of one equivalent of ketone **1** in dioxane, providing 2-bromo-1-([2.2]paracyclophan-4-yl)ethan-1-one (**2**) in 81% yield.

**Figure 1:**
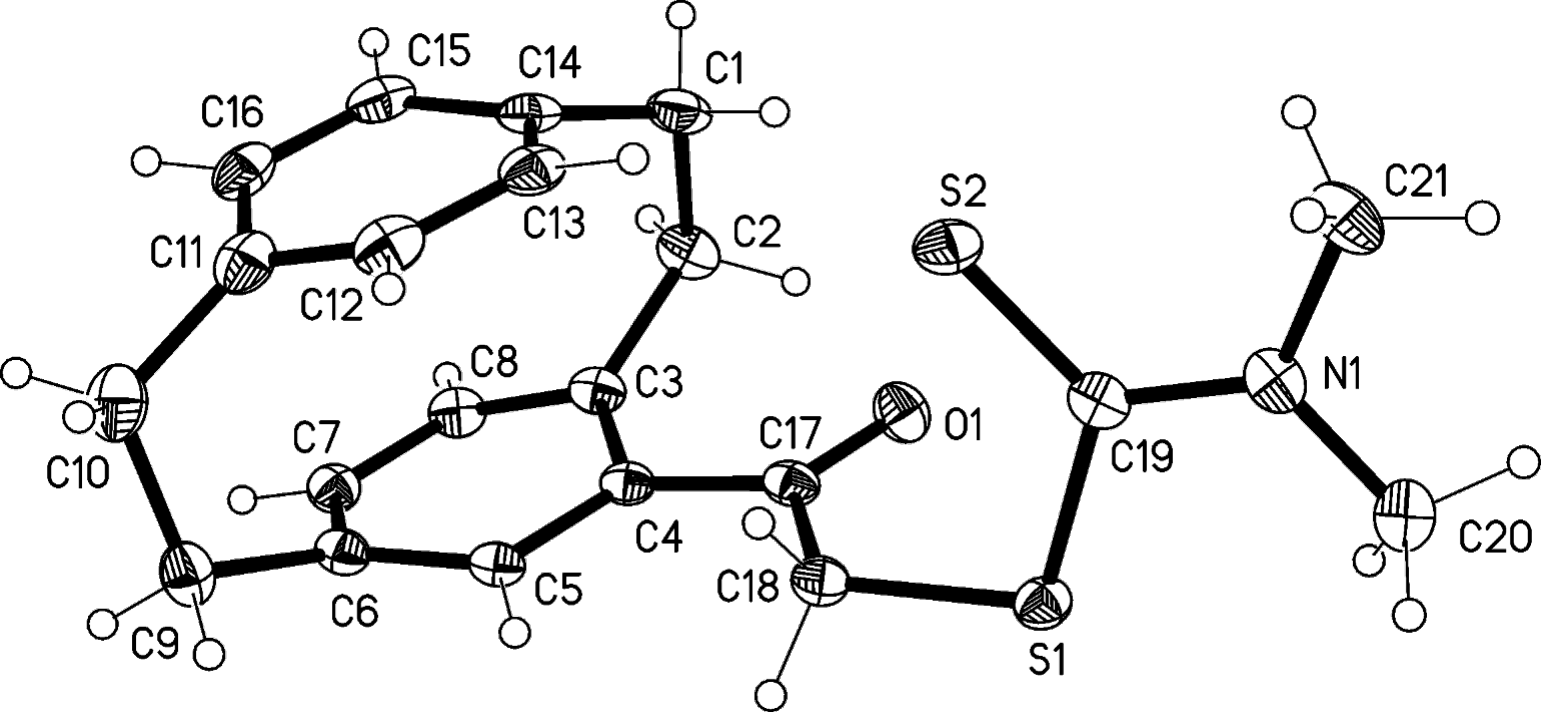
Molecular structure of compound **4a**. Ellipsoids represent 30% probability levels. Selected molecular dimensions (Å, °): N(1)–C(19) 1.341(3), N(1)–C(20) 1.458(3), N(1)–C(21) 1.471(3), N(1)–C(19)–S(2) 123.19(17), N(1)–C(19)–S(1) 113.85(17), S(2)–C(19)–S(1) 122.89(13).

The reactions of α-bromophenones with salts of dithiocarbamic acid, readily available from the reaction of secondary amines with carbon disulfide, represent an accessible route to variously substituted phenacyl carbodithioates [19]. Following this synthetic protocol, we obtained dithiocarbamates **4a**–**c** by reacting 2-bromo-1-([2.2]paracyclophan-4-yl)ethan-1-one (**2**) with sodium *N*,*N*-dimethyldithiocarbamate (**3a**), pyrrolidinium pyrolidine-1-carbodithioate (**3b**) and morpholinium morpholine-4-carbodithioate (**3c**), respectively. These compounds were obtained as colorless crystals in 80% isolated yields. The structures of dithiocarbamates **4a**–**c** were inferred from their analytical and spectral data; thus the ^1^H NMR spectra include the expected signals for the aliphatic hydrogen atoms from the dialkylamino groups, and the ^13^C NMR spectra show the signals at ca. 196 ppm attributable to the thiocarbonyl group. Finally, the structure of 2-([2.2]paracyclophan-4-yl)-2-oxoethyl-*N*,*N*-dimethyldithiocarbamate (**4a**) was unambiguously confirmed by X-ray crystallography (Figure 1). The molecular dimensions confirm the extensive p–π conjugation within the dithiocarbamate group; the N(1)–C(19) bond length is 1.341(3) Å, some 0.12 Å shorter than N(1)–C(20) and N(1)–C(21), which are essentially σ-bonds. The cyclophane group displays the usual indicators of strain (e.g., lengthened bridge bonds and widened bridge angles, flattened boat conformations for the rings). The moieties C3/4/5/17/18/O1 and C19/20/21/S1/S2/N1 are to a good approximation planar and subtend an interplanar angle of 80°. Short intramolecular contacts H13···S2 2.83 Å and H2B···O1 2.29 Å are observed. CCDC-1412356 contains the supplementary crystallographic data for compound **4a** (see also Supporting Information File 1). These data can be obtained free of charge from the Cambridge Crystallographic Data Center via http://www.ccdc.cam.ac.uk/data_request/cif.

The third step, the synthesis of 1,3-dithiol-2-ylium salts, consists of an acid-catalyzed cyclocondensation of phenacyl carbodithioates **4**. Using a concentrated sulfuric acid–glacial acetic acid mixture (1:3, v/v) [20], the cyclization of carbodithioates **4a**–**c** takes place under mild reaction conditions. After 10 min at 80 °C the homogeneous reaction mixture was cooled to room temperature and 70% HClO_4_ and water were added. Filtration and recrystallization of the precipitate gave perchlorates **5a**–**c** as colorless crystals, in 58–81% yield (Scheme 1). The heterocyclization of carbodithioates **4** is supported by their spectral data. The IR spectra revealed the disappearance of the absorption band corresponding to the carbonyl group (ca. 1680 cm^−1^) and the presence of new, strong and broad absorption bands at 1000–1100 cm^−1^, corresponding to the perchlorate anion. The ^1^H NMR spectra of 1,3-dithiol-2-ylium perchlorates indicate the absence of the AB quartet pattern of the methylene hydrogen atoms from compounds **4**. The ^13^C NMR spectra also confirm the successful synthesis of 1,3-dithiol-2-ylium salts **5** by the disappearance of the carbonyl and thiocarbonyl carbon atoms present in the carbodithioate spectra, as well as the appearance of a new signal at a very low field (ca. 180 ppm) which corresponds to the electron-deficient C-2 atom.

**Scheme 2:**
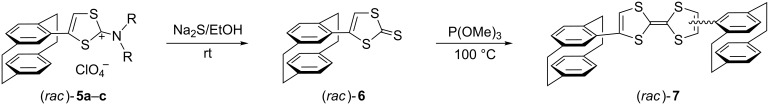
Synthesis of tetrathiafulvalenes **7**.

1,3-Dithiolium salts are valuable precursors for tetrathiafulvalenes. There are two main synthetic approaches that are mainly based on the exploitation of the electron-deficient character of the C-2 carbon atom. One of these involves the conversion of 2-*N*,*N*-dialkylamino-1,3-dithiolium salts into the corresponding 2-unsubstituted 1,3-dithiolium salts, followed by the homocoupling of the carbene intermediate that is generated under basic conditions. Unfortunately, our attempts to synthesize the 2-unsubstituted derivative from 1,3-dithiolium perchlorates **5a**–**c** in the presence of tetrafluoroboric acid led to the degradation of the substrate. The second approach for the synthesis of tetrathiafulvalenes involves the desulfurative coupling of 1,3-dithiol-2-thiones in the presence of alkyl phosphites. 1,3-Dithiol-2-thiones are easily available by the treatment of 1,3-dithiol-2-ylium salts with sodium sulfide [21]. Thus, by treating perchlorates **5a**–**c** with sodium sulfide nonahydrate, at room temperature in ethanol, we obtained 4-([2.2]paracyclophan-4-yl)-1,3-dithiol-2-thione (**6**) as a yellow solid in 41% isolated yield (Scheme 2).

**Figure 2:**
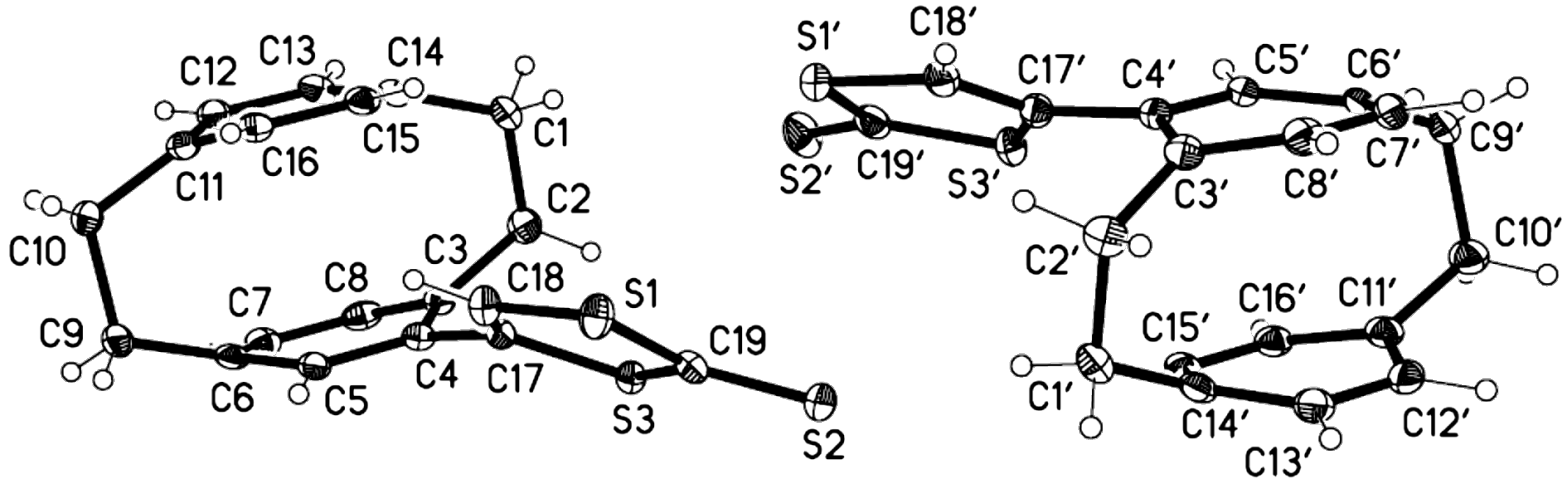
Molecular structure of compound **6** (two independent molecules). Ellipsoids represent 30% probability levels.

Unexpectedly, the yield of 1,3-dithiol-2-thione **6** was found to be dependent on the nature of the dialkylamino substituent and to the volume of the solvent used. The best results were obtained by employing 2-(*N*,*N*-dimethylamino)-4-([2.2]paracyclophan-4-yl)-1,3-dithiol-2-ylium perchlorate (**5a**) as a substrate for this reaction. The formation of 1,3-dithiol-2-thione **6** is supported by the NMR and mass spectrometry data. Moreover, the structure of 1,3-dithiol-2-thione **6** was confirmed by X-ray crystallography (Figure 2). The asymmetric unit contains two molecules, which differ in the orientation of the 1,3-dithiol-2-thione group relative to the cyclophane; the torsion angle C3–C4–C17–S3 is −45.9° in the first molecule but −142.4° in the second. CCDC-1412357 contains the supplementary crystallographic data for compound **6** (see also Supporting Information File 1). These data can be obtained free of charge from The Cambridge Crystallographic Data Center via http://www.ccdc.cam.ac.uk/data_request/cif.

The desulfurative dimerization of 1,3-dithiol-2-thione **6** was effected by heating it with trimethyl phosphite at 100 °C (Scheme 2). Purification of the crude reaction mixture by column chromatography resulted in a mixture of inseparable isomeric tetrathiafulvalenes **7** in 17% yield. Since compound **6** already exhibits planar chirality (racemate *R*_p_/*S*_p_), the theoretical number of stereoisomers of the tetrathiafulvalene **7** should be six as follows: *cis*-(*S*_p_,*S*_p_) with *cis*-(*R*_p_,*R*_p_) and *trans*-(*S*_p_,*S*_p_) with *trans*-(*R*_p_,*R*_p_) for the parallel orientation of the ethano bridges, and the mesoforms *cis*-(*S*_p_,*R*_p_) and *trans*-(*S*_p_,*R*_p_) for the angular orientation of the ethano bridges. The NMR spectrum of the purified tetrathiafulvalene indicates the presence of 4 isomers, most probably the two pairs of racemates and the two mesoforms.

Although the separation of the isomers has not yet been successful, this synthetic pathway indicates that it is feasible to incorporate of [2.2]paracyclophane as an extended π-system onto a tetrathiafulvalene core. Further research will target a decrease in the theoretical number of isomers and their isolation in order to investigate their structural and chiroptical properties.

## Conclusion

The synthesis of as yet inseparable isomeric tetrathiafulvalenes has been performed by desulfurative dimerization of a [2.2]paracyclophane-substituted trithione. The latter compound was obtained from the corresponding 1,3-dithiol-2-ylium cation. This was in turn synthesized through a three-step procedure, starting with the regioselective bromination of 4-acetyl[2.2]paracyclophane.

## Supporting Information

File 1 Detailed experimental procedures, supplementary spectroscopic and X-ray data.
