# Supplementary material for: [2.2]Paracyclophane derivatives containing tetrathiafulvalene moieties
Source: Beilstein J Org Chem. 2015 Oct 15;11:1917–21. doi: 10.3762/bjoc.11.207 (PMC4660956; doi:10.3762/bjoc.11.207)
Supplement: File 1 — Detailed experimental procedures, supplementary spectroscopic and X-ray data. [file Beilstein_J_Org_Chem-11-1917-s001.pdf]

Supporting Information

for

[2.2]Paracyclophane derivatives containing tetrathiafulvalene moieties

Laura G. Sarbu[1,2], Lucian G. Bahrin[1,2], Peter G. Jones[3], Lucian M. Birsa*[1,2], and Henning Hopf*[2]

Address: [1]Department of Chemistry, “Al. I. Cuza” University of Iasi, 11 Carol I Bv., RO-700506 Iasi, Romania, [2]Institute of Organic Chemistry, Technical University of Braunschweig, Hagenring 30, D-38106 Braunschweig, Germany and [3]Institute of Inorganic and Analytical Chemistry, Technical University of Braunschweig, Hagenring 30, D-38106 Braunschweig

Email: Lucian M. Birsa* - lbirsa@uaic.ro ; Henning Hopf - h.hopf@tu-bs.de

* Corresponding author

Detailed experimental procedures, supplementary spectroscopic and X-ray data

Table of Contents

A. Experimental section S2

B. X-ray spectral data..S8

A. Experimental section

General Remarks: Melting points: Büchi 510, uncorrected. IR: Bruker Tensor 27 with ATR. ^{1}H and ^{13}C NMR: Bruker AV300 spectrometer with TMS as internal standard at room temperature. Chemical shifts are reported in ppm downfield from tetramethylsilane. MS: Finnigan MAT 90X, electron impact (EI). Elemental analyses: CE440 Elemental Analyser; the results were found to be in good agreement (±0.30%) with the calculated values. All reagents were commercially available and used without further purification.

2-Bromo-1-([2.2]paracyclophan-4-yl)ethan-1-one (2)

To a solution of 4-acetyl[2.2]paracyclophane (**1**) [1] (4.67 g, 18.70 mmol) in dioxane (25 mL), a solution of the molecular complex Br_2·dioxane (1 mL Br_2, 1.6 mL dioxane) in dioxane (10 mL) was added dropwise. The reaction mixture was left to react at rt for 30 min and then poured in water. The precipitate was filtered off, washed with water, dried and recrystallized from ethanol to provide compound **2** as a white solid, m.p. 115-116 °C (lit. 101-103 °C [2]); yield 81% (5.0 g); IR (KBr): nu(tilde) = 2929, 2921, 2851, 1694, 1211, 993, 720, 630, 505 cm^{-1}; ^{1}H NMR ($CDCl_3$, 300 MHz): δ = 2.80–2.92 (m, 1 H, CH_2), 2.95–3.07 (m, 2 H, CH_2), 3.11–3.28 (m, 4 H, CH_2), 3.38–3.92 (m, 1 H, CH_2), 4.15, 4.36 (ABq, ^{2}J = 12.2 Hz, 2 H, CH_2), 6.38 (dd, ^{4}J = 1.8 Hz, ^{3}J = 7.9 Hz, 1 H, CH_{ar}), 6.53 (m, 4 H, CH_{ar}), 6.69 (dd, ^{4}J = 1.7 Hz, ^{3}J = 7.9 Hz, 1 H, CH_{ar}), 6.95 ppm (d, ^{4}J = 1.7 Hz, 1 H,CH_{ar}); ^{13}C NMR ($CDCl_3$, 75 MHz) δ = 33.2

1. Truesdale, E. A.; Cram, D. J. *J. Org. Chem.* **1980**, *45*, 3974-3981.
2. Mamyrbekova, Zh. A.; Soldatova, S. A.; Abelentsev, V. I.; Solov'eva, T. I.; Guryshev, V. N.; Soldatenkov, A. T. *Pharm. Chem. J.* **1994**, *28*, 198-202.

(CH_2), 34.8 (CH_2), 35.0 (CH_2), 35.1 (CH_2), 36.0 (CH_2), 131.2 (CH), 132.3 (CH), 132.7 (CH), 132.8 (CH), 133.6 (CH), 134.6 (C), 136.6 (CH), 137.3 (CH), 139.1 (C), 140.0 (C), 141.1 (C), 142.9 (C), 192.7 ppm (s); MS (EI, 70 eV): *m/z* (%) = 310 (27) [M^++2] for ^{81}Br, 328 (28) [M^+] for ^{79}Br, 249 (32), 145 (67), 104 (100).

2-([2.2]Paracyclophan-4-yl)-2-oxoethyl-*N*,*N*-dimethyldithiocarbamate (4a)

General procedure

To a solution of 2-bromo-1-([2.2]paracyclophan-4-yl)ethan-1-one (**2**, 4.29 g, 13.04 mmol) in acetone (100 mL) a solution of sodium *N*,*N*-dimethyldithiocarbamate (**3a**, 2.24 g, 13.04 mmol) in acetone/water (40 mL, 1:1 v/v) was added. The reaction mixture was heated under reflux for 10 min, cooled to rt and then poured into water. The precipitate was filtered off, washed with water, dried, and recrystallized from ethanol to provide compound **4a**. Dithiocarbamates **4b** and **4c** were obtained under the same experimental conditions.

Compound 4a: Colorless solid; m.p. 143-144 °C; yield 81% (3.72 g); IR (KBr): nu(tilde) = 2921, 2883, 2850, 1683, 1498, 1295, 978, 720, 633, 502 cm^{-1}; ^{1}H NMR ($CDCl_3$, 300 MHz): δ = 2.77–2.89 (m, 1 H, CH_2), 2.95–3.33 (m, 6 H, CH_2), 3.50 (s, 3 H, CH_3), 3.59 (s, 3 H, CH_3), 3.77–3.88 (m, 1 H, CH_2), 4.22, 5.01 (ABq, ^2J = 17.2 Hz, 2 H, CH_2), 6.41–6.45 (m, 1 H, CH_{ar}), 6.48–6.60 (m, 4 H, CH_{ar}), 6.70 (dd, ^4J = 1.7 Hz, ^3J = 7.8 Hz, 1 H, CH_{ar}), 7.30 ppm (d, ^4J = 1.7 Hz, 1 H, CH_{ar}); ^{13}C NMR ($CDCl_3$, 75 MHz): δ = 34.9 (CH_2), 35.0 (CH_2), 35.1 (CH_2), 35.9 (CH_2), 41.7 (CH_3), 45.8 (CH_3), 46.7 (CH_2), 131.5 (CH), 132.5 (CH), 132.6 (CH), 132.7 (CH), 134.0 (CH), 136.2 (C), 136.3 (CH), 136.9 (CH), 139.1 (C), 140.0 (C), 140.1 (C), 142.0 (C), 194.6 (C), 195.9 ppm (C); MS (EI, 70 eV): *m/z* (%) = 369 (23) [M^+], 354 (5), 131 (9), 104 (12), 88 (100).

Compound 4b: Colorless solid; m.p. 104-105 °C; yield 80% (0.89 g); IR (KBr): nu(tilde) = 2926, 2871, 1681, 1430, 1158, 955, 720, 632 cm^{-1}; ^1H NMR ($CDCl_3$, 300 MHz): δ = 1.85–2.19 (m, 4 H, CH_2), 2.70–3.40 (m, 8 H, CH_2), 3.61–4.03 (m, 4 H, CH_2), 4.22, 5.01 (ABq, ^2J = 17.0 Hz, 2 H, CH_2), 6.30–6.82 ppm (m, 7 H, CH_{ar}); ^{13}C NMR ($CDCl_3$, 75 MHz): δ = 24.0 (CH_2), 25.8 (CH_2), 34.5 (CH_2), 34.7 (CH_2), 34.8 (CH_2), 35.5 (CH_2), 45.4 (CH_2), 50.4 (CH_2), 55.0 (CH_2), 131.1 (CH), 132.1 (CH), 132.3 (CH), 132.4 (CH), 133.6 (CH), 135.8 (C), 135.9 (CH), 136.5 (CH), 138.7 (C), 139.6 (C), 139.7 (C), 141.5 (C), 190.1 (C), 194.3 ppm (C); MS (EI, 70 eV)): *m/z* (%) = 395 (20) [M^+], 146 (21), 114 (100), 104 (33).

Compound 4c: White solid; m.p. 105-106 °C; yield 80% (0.56 g); IR 3011, 2925, 2853, 1688, 1425, 1228, 1112, 990, 723, 634, 540 cm^{-1}; ^1H NMR ($CDCl_3$, 300 MHz) δ 2.77–2.90 (m, 1 H, CH_2), 2.93–3.33 (m, 7 H, CH_2), 3.77–3.85 (m, 4 H, CH_2), 3.93–4.51 (m, 4 H, CH_2), 4.27, 5.01 (ABq, ^2J = 17.1 Hz, 2 H, CH_2), 6.42 (dd, ^4J = 1.9 Hz, ^3J = 8.6 Hz, 1 H, CH_{ar}); 6,48–6,58 (m, 4 H, CH_{ar}); 6,70 (dd, ^4J= 1.8 Hz; ^3J = 7.8 Hz, 1 H, CH_{ar}); 7.26 (d, ^4J = 1.8 Hz, 1H, CH_{ar}) ppm; ^{13}C NMR ($CDCl_3$, 75 MHz) δ 35.0 (CH_2), 35.1 (2xCH_2), 35.9 (CH_2), 62.1 (CH_2), 62.2 (3xCH_2), 46.0 (CH_2), 131.4 (CH), 132.4 (CH), 132.7 (CH), 132.8 (CH), 133.9 (CH), 136.2 (C), 136.3 (CH), 136.9 (CH), 139.1 (C), 140.0 (C), 140.1 (C), 142.1 (C), 194.4 (C), 196.3 (s) ppm; MS (EI) *m/z* (%) 411 (25) [M^+], 130 (100), 104 (24), 86 (30).

2-(*N*,*N*-Dimethylamino)-4-([2.2]paracyclophan-4-yl)-1,3-dithiol-2-ylium perchlorate (5a)

General procedure

To a mixture of 6 mL concentrated sulfuric acid and 18 mL glacial acetic acid, 2-([2.2]paracyclophan-4-yl)-2-oxoethyl-*N*,*N*-dimethyldithiocarbamate (**4a**, 2.74 g, 7.42

mmol) was added in small portions. The reaction mixture was heated at 80 °C for 10 min, cooled after which 3 mL of perchloric acid were added. The precipitate obtained after water addition (250 mL) was filtered, dried and recrystallized from ethanol to provide compound **5a**. 1,3-Dithiolium perchlorates **5b** and **5c** were obtained under the same experimental conditions.

Compound 5a: White solid; m.p. 260-261 °C; yield 63% (2.11 g); IR 3049, 2928, 1576, 1404, 1074, 720, 619, 512 cm^{-1}; ^1H NMR (DMSO-*d*6, 300 MHz) δ 2.79–2.91 (m, 1 H, CH_2), 2.96–3.13 (m, 6 H, CH_2), 3.41–3.52 (m, 1 H, CH_2), 3.56 (s, 3 H, CH_3), 3.60 (s, 3 H, CH_3), 6.55 (dd, ^4J = 1.5 Hz, ^3J = 7.8 Hz, 1 H, CH_{ar}), 6.58–6.72 (m, 6 H, CH_{ar}), 7.69 (s, 1 H, CH) ppm; ^{13}C NMR (DMSO-*d*6, 75 MHz) δ 33.5 (CH_2), 34.1 (CH_2), 34.2 (CH_2), 34.6 (CH_2), 46.6 (CH_3), 47.6 (CH_3), 120.8 (CH), 129.5 (CH), 129.7 (C), 131.9 (CH), 132.7 (CH), 132.9 (CH), 133.3 (CH), 134.9 (CH), 136.4 (CH), 138.0 (C), 138.7 (C), 138.8 (C), 139.4 (C), 141.0 (C), 187.0 (C) ppm; MS (ESI) *m/z* (%) 352 [M^+ - ClO_4].

Compound 5b: White solid; m.p. 235-236 °C; yield 58% (0.62 g); IR 2931, 2857, 1579, 1439, 1257, 1077, 718, 620 cm^{-1}; ^1H NMR (DMSO-*d*6, 300 MHz) δ 2.17–2.28 (m, 4 H, CH_2), 2.76–2.91 (m, 1 H, CH_2), 2.95–3.16 (m, 6 H, CH_2), 3.38–3.55 (m, 1 H, CH_2), 3.71–3.92 (m, 4 H, CH_2), 6.55 (dd, ^4J = 1.5 Hz, ^3J = 7.8 Hz, 1 H, CH_{ar}), 6.58–6.74 (m, 6 H, CH_{ar}), 7.67 (s, 1 H, CH) ppm; ^{13}C NMR (DMSO-*d*6, 75 MHz) δ 26.1 (CH_2), 26.2 (CH_2), 33.5 (CH_2), 34.1 (CH_2), 34.2 (CH_2), 34.6 (CH_2), 56.4 (CH_2), 57.3 (CH_2), 120.6 (CH), 129.4 (CH), 129.7 (C), 131.9 (CH), 132.7 (CH), 132.9 (CH), 133.3 (CH), 134.9 (CH), 136.4 (CH), 138.0 (C), 138.4 (C), 138.8 (C), 139.4 (C), 141.0 (C), 181.3 (C) ppm; MS (ESI) *m/z* (%) 378 [M^+ - ClO_4].

Compound 5c: White solid; m.p. 213-214 °C; yield 81% (0.24 g); IR 2918, 2849, 1576, 1431, 1258, 1077, 719, 620, 540 cm^{-1}; ^1H NMR (DMSO-*d*6, 300 MHz) δ 2.80–2.93 (m, 1 H, CH_2), 2.94–3.14 (m, 6 H, CH_2), 3.40–3.50 (m, 1 H, CH_2), 3.84–3.92 (m,

4 H, CH_2), 3.92–4.00 (m, 4 H, CH_2), 6.55 (dd, ^4J = 1.4 Hz, ^3J = 7.7 Hz, 1 H, CH_{ar}), 6.57–6.74 (m, 6 H, CH_{ar}), 7.71 (s, 1 H, CH) ppm; ^{13}C NMR (DMSO-*d*6, 75 MHz) δ 33.4 (CH_2), 34.1 (CH_2), 34.2 (CH_2), 34.6 (CH_2), 53.4 (CH_2), 54.6 (CH_2), 64.3 (2xCH_2), 119.8 (CH), 129.4 (CH), 129.6 (C), 131.9 (CH), 132.7 (CH), 132.8 (CH), 133.3 (CH), 135.0 (CH), 136.4 (CH), 137.6 (C), 138.0 (C), 138.8 (C), 139.3 (C), 141.0 (C), 188.1 (s) ppm; MS (ESI) *m/z* (%) 394 [M^+ - ClO_4].

4-([2.2]Paracyclophan-4-yl)-1,3-dithiol-2-thione (6)

To a suspension of 2-(*N*,*N*-dimethylamino)-4-([2.2]paracyclophan-4-yl)-1,3-dithiol-2-ylium perchlorate (**5a**, 2.18 g, 4.82 mmol) in ethanol (100 mL) $Na_2S·9H_2O$ (2.32 g, 9.64 mmol) was added. The reaction mixture was allowed to react for 12 h at rt. The precipitate obtained after water addition (300 mL) was filtered, dried and purified by column chromatography using CH_2Cl_2/pentane (1:1) as eluent to provide compound **6** as a yellow solid. M.p. 137-138 °C; R_f = 0.49; yield 41% (0.67 g); IR 2922, 2849, 1054, 799, 718, 645 cm^{-1}; ^{1}H NMR ($CDCl_3$, 300 MHz) δ 2.81–2.89 (m, 1 H, CH_2), 2.89–3.09 (m, 6 H, CH_2), 3.36–3.49 (m, 1 H, CH_2), 6.32–6.38 (m, 2 H, CH_{ar}), 6.48–6.58 (m, 4 H, CH_{ar}), 6.61 (dd, ^4J = 1.8 Hz, ^3J = 7.9 Hz, 1 H, CH_{ar}), 6.75 (s, 1 H, CH) ppm; ^{13}C NMR ($CDCl_3$, 75 MHz) δ 33.8 (CH_2), 34.6 (CH_2), 34.7 (CH_2), 35.0 (CH_2), 124.3 (CH), 129.5 (CH), 130.9 (C), 131.7 (CH), 132.3 (CH), 132.5 (CH), 133.0 (CH), 134.2 (CH), 136.0 (CH), 137.5 (C), 138.9 (C), 139.2 (C), 140.4 (C), 146.2 (C), 212.7 (C) ppm; MS (EI) *m/z* (%) 340 (52) [M^+], 264 (16), 160 (100), 104 (48).

Tetrathiafulvalenes 7

A mixture of 4-([2.2]paracyclophan-4-yl)-1,3-dithiol-2-thione (**6**, 0.150 g, 0.44 mmol) and trimethyl phosphite (0.52 mL, 4.4 mmol) was heated at 100 °C for 14 h. The reaction mixture was purified by column chromatography using CH_2Cl_2/pentane (1:1)

as eluent to provide tetrathiafulvalenes **7** as yellow solids. M.p. 137-138 °C; R_f = 0.60; yield 17% (0.023 g); IR 2922, 2889, 2850, 1587, 1499, 939, 720, 644 cm^{-1}; ^{1}H NMR ($CDCl_3$, 300 MHz) selected data for the major isomer δ 2.96–3.10 (m, 14 H, CH_2), 3.66 (m, 2 H, CH_2), 6.29–6.63 (m, 14 H, CH_{ar}), 6.79 (m, 2 H, CH) ppm; ^{13}C NMR ($CDCl_3$, 75 MHz) selected data for the major isomer δ 35.1 (CH_2), 35.2 (CH_2), 35.4 (CH_2), 35.5 (CH_2), 132.3 (CH_2), 132.4 (CH_2), 132.6 (CH_2), 132.7 (CH_2), 133.1 (CH_2), 135.9 (C), 136.0 (C), 139.3 (C), 139.4 (C), 139.5 (C), 139.6 (C) ppm; MS (EI) *m/z* (%) 616 (77) [M^+], 583 (15), 160 (62), 128 (100), 104 (60).

B. X-ray spectral data

X-ray structure determinations

Crystal data are summarized in Table S1. Crystals were mounted in inert oil on glass fibers and transferred to the cold gas stream of an Oxford Diffraction Nova E diffractometer. Intensity measurements were performed using mirror-focussed CuKα radiation (λ = 1.54184 Å). Absorption corrections were based on multi-scans. Structures were refined anisotropically on F^2 using the program SHELXL-97 [3]. Hydrogen atoms were included using a riding model or rigid methyl groups. Compound **6** was not enantiomerically pure and crystallized only by chance in the chiral (Sohncke) space group $P2_1$; the structure was refined as an enantiomeric twin, with a Flack parameter of 0.440(16).

3. Sheldrick, G. M. *Acta Cryst*. **2008**, *A64*, 112-122.

Table S1: Crystallographic data for **4a** and **6**

Compound	**4a**	**6**
Formula	$C_{21}H_{23}NOS_2$	$C_{19}H_{16}S_3$
M_r	369.52	340.50
Temperature (K)	100	100
Crystal habit	colourless plate	yellow prism
Crystal size (mm)	0.1 × 0.1 × 0.015	0.15 × 0.08 × 0.04
Crystal system	orthorhombic	Monoclinic
Space group	*Pbca*	$P2_1$
Cell dimensions:		
a (Å)	11.8026(7)	7.4723(3)
b (Å)	8.2761(4)	23.1083(8)
c (Å)	37.697(3)	9.2378(4)
α (°)	90	90
β (°)	90	93.560(3)
γ (°)	90	90
Cell volume ($Å^3$)	3682.2	1592.04
Z	8	4
D_x ($g\ cm^{-3}$)	1.333	1.421
μ (mm^{-1})	2.68	4.18
2θ(max) (°)	155.2	151.2
Reflections collected	75990	32381
Independent reflections	3833	6557
R(int)	0.078	0.043
Transmissions	0.607 - 1.000	0.630 - 1.000
Data/restraints/parameters	3833/0/228	6557/1/398
Goodness-of-fit on F^2	1.09	1.07
*wR*2 (all reflections)	0.116	0.121
$R1$ ($F > 4\sigma(F)$)	0.048	0.045
Max. Δρ ($e\ Å^{-3}$)	0.62	0.70

B1. X-ray data for 2-([2.2]paracyclophan-4-yl)-2-oxoethyl-*N*,*N*-dimethyldithiocarbamate (4a)

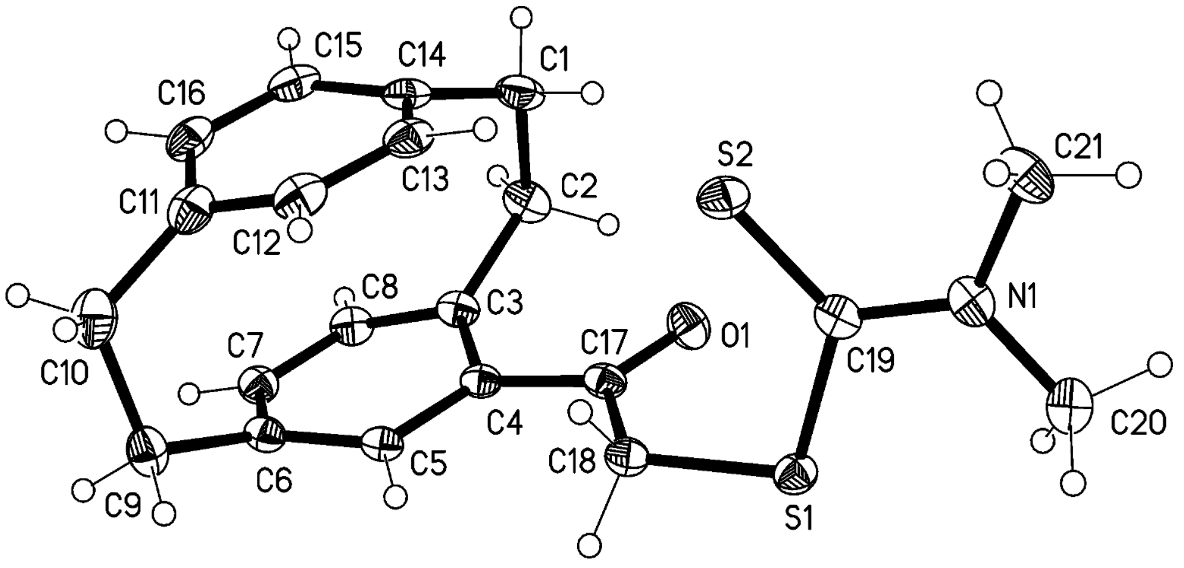

Table S2: Crystal data and structure refinement.

Identification code	perc	
Empirical formula	$C_{21}H_{23}NOS_2$	
Formula weight	369.52	
Temperature	100(2) K	
Wavelength	1.54184 Å	
Crystal system	Orthorhombic	
Space group	Pbca	
Unit cell dimensions	a = 11.8026(7) Å	α= 90°
	b = 8.2761(4) Å	β= 90°
	c = 37.697(3) Å	γ = 90°
Volume	3682.2(4) $Å^3$	
Z	8	
Density (calculated)	1.333 Mg/m^3	
Absorption coefficient	2.677 mm^{-1}	
F(000)	1568	
Crystal size	0.10 x 0.10 x 0.02 mm^3	
Theta range for data collection	4.42 to 77.61°	
Index ranges	-14<=h<=14, -9<=k<=10, -46<=l<=47	
Reflections collected	75990	
Independent reflections	3833 [R(int) = 0.0781]	
Completeness to theta = 75.00°	100.0 %	
Absorption correction	Semi-empirical from equivalents	
Max. and min. transmission	1.00000 and 0.60742	
Refinement method	Full-matrix least-squares on F^2	
Data / restraints / parameters	3833 / 0 / 228	
Goodness-of-fit on F^2	1.090	
Final R indices [I>2sigma(I)]	R1 = 0.0478, wR2 = 0.1116	
R indices (all data)	R1 = 0.0548, wR2 = 0.1162	
Largest diff. peak and hole	0.618 and -0.387 e.$Å^{-3}$	

Table S3: Atomic coordinates (x 10^4) and equivalent isotropic displacement parameters ($Å^2$x 10^3). U(eq) is defined as one third of the trace of the orthogonalized U_{ij} tensor.

	x	y	z	U(eq)
S(1)	2938.6(5)	814.7(6)	500.2(1)	31.4(1)
S(2)	4769.9(5)	-1147.2(7)	858.8(2)	37.5(2)
O(1)	4721.2(14)	3127(2)	684.1(4)	39.3(4)
N(1)	4089.5(17)	-1500(2)	193.0(5)	33.8(4)
C(1)	6822.5(19)	3332(3)	1226.2(7)	43.0(6)
C(2)	6211.5(19)	4933(3)	1110.0(7)	39.1(5)
C(3)	5075.9(18)	5137(3)	1291.5(6)	30.1(4)
C(4)	4148.3(17)	4080(2)	1249.4(6)	26.9(4)
C(5)	3385.8(17)	3887(3)	1532.5(6)	28.5(4)
C(6)	3515.4(18)	4732(3)	1849.3(6)	31.0(5)
C(7)	4266.0(19)	6025(3)	1850.2(6)	32.7(5)
C(8)	5027.4(19)	6224(3)	1575.4(6)	33.1(5)
C(9)	3066(2)	4042(3)	2192.6(6)	40.5(5)
C(10)	3967(3)	2986(3)	2398.6(7)	48.1(6)
C(11)	4980(2)	2549(3)	2173.2(6)	39.7(5)
C(12)	4931(2)	1399(3)	1903.4(7)	39.2(5)
C(13)	5654(2)	1498(3)	1613.7(7)	37.7(5)
C(14)	6446.9(18)	2747(3)	1586.2(7)	36.9(5)
C(15)	6659(2)	3643(3)	1893.1(7)	41.1(6)
C(16)	5925(2)	3544(3)	2179.8(7)	41.3(6)
C(17)	4058.7(18)	3024(3)	931.3(6)	29.6(4)
C(18)	3105.3(18)	1782(3)	921.6(6)	30.8(4)
C(19)	3999.6(18)	-700(3)	500.9(6)	30.1(4)
C(20)	3403(2)	-1130(3)	-118.3(6)	39.7(5)
C(21)	4837(2)	-2917(3)	167.9(7)	43.7(6)

Table S4: Bond lengths [Å] and angles [°].

S(1)-C(19)	1.772(2)	C(5)-C(6)	1.393(3)
S(1)-C(18)	1.790(2)	C(6)-C(7)	1.389(3)
S(2)-C(19)	1.669(2)	C(6)-C(9)	1.511(3)
O(1)-C(17)	1.219(3)	C(7)-C(8)	1.381(3)
N(1)-C(19)	1.341(3)	C(9)-C(10)	1.581(4)
N(1)-C(20)	1.458(3)	C(10)-C(11)	1.511(4)
N(1)-C(21)	1.471(3)	C(11)-C(16)	1.387(4)
C(1)-C(14)	1.508(4)	C(11)-C(12)	1.394(4)
C(1)-C(2)	1.570(4)	C(12)-C(13)	1.388(4)
C(2)-C(3)	1.514(3)	C(13)-C(14)	1.398(3)
C(3)-C(8)	1.399(3)	C(14)-C(15)	1.397(3)
C(3)-C(4)	1.410(3)	C(15)-C(16)	1.387(4)
C(4)-C(5)	1.405(3)	C(17)-C(18)	1.524(3)
C(4)-C(17)	1.488(3)		
C(19)-S(1)-C(18)	103.75(10)	C(11)-C(10)-C(9)	112.9(2)
C(19)-N(1)-C(20)	123.27(19)	C(16)-C(11)-C(12)	116.9(2)
C(19)-N(1)-C(21)	119.8(2)	C(16)-C(11)-C(10)	119.0(2)
C(20)-N(1)-C(21)	116.7(2)	C(12)-C(11)-C(10)	122.8(2)
C(14)-C(1)-C(2)	112.8(2)	C(13)-C(12)-C(11)	120.6(2)
C(3)-C(2)-C(1)	111.99(19)	C(12)-C(13)-C(14)	120.9(2)
C(8)-C(3)-C(4)	116.9(2)	C(15)-C(14)-C(13)	116.8(2)
C(8)-C(3)-C(2)	117.0(2)	C(15)-C(14)-C(1)	121.5(2)
C(4)-C(3)-C(2)	124.5(2)	C(13)-C(14)-C(1)	120.1(2)
C(5)-C(4)-C(3)	118.8(2)	C(16)-C(15)-C(14)	120.2(2)
C(5)-C(4)-C(17)	119.98(19)	C(11)-C(16)-C(15)	121.5(2)
C(3)-C(4)-C(17)	120.68(19)	O(1)-C(17)-C(4)	122.0(2)
C(6)-C(5)-C(4)	121.6(2)	O(1)-C(17)-C(18)	120.1(2)
C(7)-C(6)-C(5)	117.3(2)	C(4)-C(17)-C(18)	117.89(18)
C(7)-C(6)-C(9)	120.9(2)	C(17)-C(18)-S(1)	113.83(15)
C(5)-C(6)-C(9)	120.4(2)	N(1)-C(19)-S(2)	123.19(17)
C(8)-C(7)-C(6)	120.3(2)	N(1)-C(19)-S(1)	113.85(17)
C(7)-C(8)-C(3)	121.6(2)	S(2)-C(19)-S(1)	122.89(13)
C(6)-C(9)-C(10)	113.2(2)		

Table S5: Torsion angles [°].

C(14)-C(1)-C(2)-C(3)	-24.8(3)	O(1)-C(17)-C(18)-S(1)	-9.9(3)
C(1)-C(2)-C(3)-C(8)	102.4(2)	C(4)-C(17)-C(18)-S(1)	170.39(15)
C(1)-C(2)-C(3)-C(4)	-62.9(3)	C(19)-S(1)-C(18)-C(17)	80.88(16)
C(8)-C(3)-C(4)-C(5)	-15.5(3)	C(20)-N(1)-C(19)-S(2)	178.90(17)
C(2)-C(3)-C(4)-C(5)	149.8(2)	C(21)-N(1)-C(19)-S(2)	4.4(3)
C(8)-C(3)-C(4)-C(17)	172.52(19)	C(20)-N(1)-C(19)-S(1)	1.8(3)
C(2)-C(3)-C(4)-C(17)	-22.1(3)	C(21)-N(1)-C(19)-S(1)	-172.69(16)
C(3)-C(4)-C(5)-C(6)	1.1(3)	C(18)-S(1)-C(19)-N(1)	-174.77(16)
C(17)-C(4)-C(5)-C(6)	173.13(19)	C(18)-S(1)-C(19)-S(2)	8.12(16)
C(4)-C(5)-C(6)-C(7)	13.8(3)		
C(4)-C(5)-C(6)-C(9)	-152.8(2)		
C(5)-C(6)-C(7)-C(8)	-14.1(3)		
C(9)-C(6)-C(7)-C(8)	152.4(2)		
C(6)-C(7)-C(8)-C(3)	-0.5(3)		
C(4)-C(3)-C(8)-C(7)	15.5(3)		
C(2)-C(3)-C(8)-C(7)	-151.0(2)		
C(7)-C(6)-C(9)-C(10)	-74.9(3)		
C(5)-C(6)-C(9)-C(10)	91.3(3)		
C(6)-C(9)-C(10)-C(11)	-13.9(3)		
C(9)-C(10)-C(11)-C(16)	92.4(3)		
C(9)-C(10)-C(11)-C(12)	-73.6(3)		
C(16)-C(11)-C(12)-C(13)	-14.2(3)		
C(10)-C(11)-C(12)-C(13)	152.0(2)		
C(11)-C(12)-C(13)-C(14)	0.2(3)		
C(12)-C(13)-C(14)-C(15)	14.4(3)		
C(12)-C(13)-C(14)-C(1)	-151.2(2)		
C(2)-C(1)-C(14)-C(15)	-64.6(3)		
C(2)-C(1)-C(14)-C(13)	100.3(3)		
C(13)-C(14)-C(15)-C(16)	-15.0(3)		
C(1)-C(14)-C(15)-C(16)	150.4(2)		
C(12)-C(11)-C(16)-C(15)	13.7(3)		
C(10)-C(11)-C(16)-C(15)	-153.1(2)		
C(14)-C(15)-C(16)-C(11)	0.9(4)		
C(5)-C(4)-C(17)-O(1)	-178.2(2)		
C(3)-C(4)-C(17)-O(1)	-6.3(3)		
C(5)-C(4)-C(17)-C(18)	1.6(3)		
C(3)-C(4)-C(17)-C(18)	173.48(18)		

B2. X-ray data for 4-([2.2]paracyclophan-4-yl)-1,3-dithiol-2-thione (6)

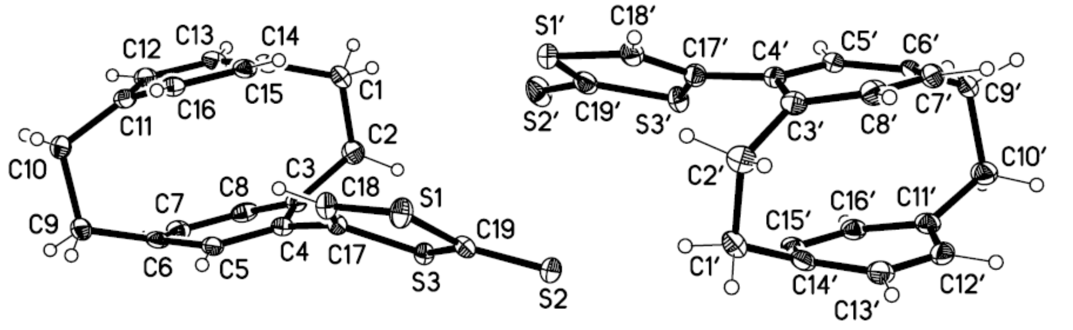

Table S6: Crystal data and structure refinement.

Identification code	dekel	
Empirical formula	$C_{19}H_{16}S_3$	
Formula weight	340.50	
Temperature	100(2) K	
Wavelength	1.54184 Å	
Crystal system	Monoclinic	
Space group	$P2_1$	
Unit cell dimensions	a = 7.4723(3) Å	α= 90°
	b = 23.1083(8) Å	β= 93.560(3)°
	c = 9.2378(4) Å	γ = 90°
Volume	1592.04(11) $Å^3$	
Z	4	
Density (calculated)	1.421 Mg/m^3	
Absorption coefficient	4.178 mm^{-1}	
F(000)	712	
Crystal size	0.15 x 0.08 x 0.04 mm^3	
Theta range for data collection	3.83 to 75.60°	
Index ranges	-9<=h<=8, -28<=k<=28, -11<=l<=11	
Reflections collected	32381	
Independent reflections	6557 [R(int) = 0.0429]	
Completeness to theta = 75.00°	99.8 %	
Absorption correction	Semi-empirical from equivalents	
Max. and min. transmission	1.00000 and 0.62972	
Refinement method	Full-matrix least-squares on F^2	
Data / restraints / parameters	6557 / 1 / 398	
Goodness-of-fit on F^2	1.071	
Final R indices [I>2sigma(I)]	R1 = 0.0450, wR2 = 0.1203	
R indices (all data)	R1 = 0.0457, wR2 = 0.1208	
Absolute structure parameter	0.440(16)	
Largest diff. peak and hole	0.696 and -0.410 e.$Å^{-3}$	

Table S7: Atomic coordinates (x 10^4) and equivalent isotropic displacement parameters ($Å^2$x 10^3). U(eq) is defined as one third of the trace of the orthogonalized U_{ij} tensor.

	x	y	z	U(eq)
S(1)	7667.7(12)	4795.7(4)	4645.2(10)	35.8(2)
S(2)	6709.8(13)	5472.1(4)	7242.1(10)	39.5(2)
S(3)	4289.7(10)	4581.0(3)	5856.6(8)	28.7(2)
C(1)	4633(5)	2790.9(18)	6002(4)	36.2(8)
C(2)	2948(5)	3200.6(16)	6006(4)	33.8(7)
C(3)	2311(4)	3406.8(14)	4496(3)	26.7(6)
C(4)	3301(4)	3789.9(14)	3656(4)	25.8(6)
C(5)	3127(4)	3760.9(14)	2143(4)	27.7(6)
C(6)	2024(4)	3350.2(14)	1424(4)	28.6(7)
C(7)	776(4)	3069.5(15)	2253(4)	32.9(7)
C(8)	929(4)	3097.1(15)	3748(4)	34.3(7)
C(9)	2421(5)	3140.9(15)	-60(4)	33.1(7)
C(10)	3755(5)	2604.9(15)	-25(4)	32.4(7)
C(11)	4497(4)	2456.5(14)	1489(4)	28.9(7)
C(12)	3556(5)	2086.8(15)	2362(4)	31.0(7)
C(13)	3777(5)	2133.4(15)	3870(4)	32.0(7)
C(14)	4932(5)	2544.7(15)	4519(4)	29.9(7)
C(15)	6131(4)	2813.5(14)	3645(4)	29.3(7)
C(16)	5916(4)	2769.2(15)	2138(4)	28.4(6)
C(17)	4698(4)	4178.4(13)	4311(3)	25.8(6)
C(18)	6288(5)	4291.5(16)	3777(4)	31.8(7)
C(19)	6253(5)	4978.5(15)	6012(4)	31.2(7)
S(1')	10343.1(13)	4310.1(4)	8064.9(9)	37.7(2)
S(2')	6837.6(16)	3879.0(5)	9032.9(12)	49.0(3)
S(3')	9073.4(11)	4739.7(4)	10733.2(8)	30.3(2)
C(1')	11040(5)	6443.4(17)	9103(4)	34.4(7)
C(2')	12889(5)	6114.4(16)	9297(4)	34.9(7)
C(3')	13214(4)	5842.0(15)	10779(4)	28.4(7)
C(4')	12181(4)	5384.7(14)	11304(3)	25.9(6)
C(5')	11944(4)	5348.4(14)	12789(3)	24.8(6)
C(6')	12735(4)	5746.4(14)	13769(3)	25.6(6)
C(7')	14066(4)	6095.2(16)	13280(4)	32.6(7)
C(8')	14296(4)	6141.7(16)	11807(4)	33.2(7)
C(9')	11908(5)	5869.6(15)	15201(4)	30.8(7)

C(10')	10613(5)	6414.0(18)	15115(4)	38.3(8)
C(11')	10114(4)	6601.4(15)	13577(4)	31.3(7)
C(12')	11084(5)	7030.6(15)	12914(4)	32.9(7)
C(13')	11213(5)	7044.9(15)	11421(4)	33.9(7)
C(14')	10354(4)	6624.9(15)	10547(4)	29.6(7)
C(15')	9043(4)	6284.4(14)	11185(4)	29.4(7)
C(16')	8926(4)	6272.2(14)	12671(4)	30.3(7)
C(17')	11188(4)	4987.3(14)	10300(3)	27.0(6)
C(18')	11758(5)	4775.6(17)	9052(3)	33.3(7)
C(19')	8643(5)	4282.5(16)	9273(4)	35.5(7)

Table S8: Bond lengths [Å] and angles [°].

S(1)-C(18)	1.720(4)	S(1')-C(18')	1.728(4)
S(1)-C(19)	1.748(4)	S(1')-C(19')	1.743(4)
S(2)-C(19)	1.631(4)	S(2')-C(19')	1.644(4)
S(3)-C(19)	1.730(4)	S(3')-C(19')	1.728(4)
S(3)-C(17)	1.747(3)	S(3')-C(17')	1.750(3)
C(1)-C(14)	1.512(5)	C(1')-C(14')	1.518(5)
C(1)-C(2)	1.575(5)	C(1')-C(2')	1.577(5)
C(2)-C(3)	1.521(5)	C(2')-C(3')	1.513(5)
C(3)-C(8)	1.403(5)	C(3')-C(8')	1.393(5)
C(3)-C(4)	1.416(4)	C(3')-C(4')	1.412(5)
C(4)-C(5)	1.398(5)	C(4')-C(5')	1.397(4)
C(4)-C(17)	1.478(4)	C(4')-C(17')	1.472(5)
C(5)-C(6)	1.398(4)	C(5')-C(6')	1.396(4)
C(6)-C(7)	1.402(5)	C(6')-C(7')	1.378(5)
C(6)-C(9)	1.501(5)	C(6')-C(9')	1.521(4)
C(7)-C(8)	1.379(5)	C(7')-C(8')	1.386(5)
C(9)-C(10)	1.589(5)	C(9')-C(10')	1.586(5)
C(10)-C(11)	1.511(5)	C(10')-C(11')	1.510(5)
C(11)-C(16)	1.389(5)	C(11')-C(12')	1.393(5)
C(11)-C(12)	1.395(5)	C(11')-C(16')	1.405(5)
C(12)-C(13)	1.398(5)	C(12')-C(13')	1.389(5)
C(13)-C(14)	1.394(5)	C(13')-C(14')	1.394(5)
C(14)-C(15)	1.390(5)	C(14')-C(15')	1.413(5)
C(15)-C(16)	1.395(5)	C(15')-C(16')	1.382(5)
C(17)-C(18)	1.340(5)	C(17')-C(18')	1.346(5)
C(18)-S(1)-C(19)	97.40(17)	C(5)-C(6)-C(7)	116.6(3)
C(19)-S(3)-C(17)	99.21(16)	C(5)-C(6)-C(9)	120.6(3)
C(14)-C(1)-C(2)	113.0(3)	C(7)-C(6)-C(9)	121.7(3)
C(3)-C(2)-C(1)	113.0(3)	C(8)-C(7)-C(6)	120.6(3)
C(8)-C(3)-C(4)	116.2(3)	C(7)-C(8)-C(3)	122.0(3)
C(8)-C(3)-C(2)	118.1(3)	C(6)-C(9)-C(10)	113.1(3)
C(4)-C(3)-C(2)	123.6(3)	C(11)-C(10)-C(9)	112.9(3)
C(5)-C(4)-C(3)	120.0(3)	C(16)-C(11)-C(12)	117.9(3)
C(5)-C(4)-C(17)	117.2(3)	C(16)-C(11)-C(10)	120.5(3)
C(3)-C(4)-C(17)	122.4(3)	C(12)-C(11)-C(10)	120.3(3)
C(4)-C(5)-C(6)	121.5(3)	C(11)-C(12)-C(13)	119.8(3)

C(14)-C(13)-C(12)	120.9(3)	C(7')-C(6')-C(5')	117.2(3)
C(15)-C(14)-C(13)	117.3(3)	C(7')-C(6')-C(9')	121.1(3)
C(15)-C(14)-C(1)	119.7(3)	C(5')-C(6')-C(9')	120.6(3)
C(13)-C(14)-C(1)	121.6(3)	C(6')-C(7')-C(8')	120.2(3)
C(14)-C(15)-C(16)	120.6(3)	C(7')-C(8')-C(3')	121.9(3)
C(11)-C(16)-C(15)	120.5(3)	C(6')-C(9')-C(10')	112.6(3)
C(18)-C(17)-C(4)	126.0(3)	C(11')-C(10')-C(9')	112.9(3)
C(18)-C(17)-S(3)	113.6(3)	C(12')-C(11')-C(16')	116.8(3)
C(4)-C(17)-S(3)	120.2(2)	C(12')-C(11')-C(10')	120.8(3)
C(17)-C(18)-S(1)	118.7(3)	C(16')-C(11')-C(10')	121.0(3)
S(2)-C(19)-S(3)	124.7(2)	C(13')-C(12')-C(11')	121.6(3)
S(2)-C(19)-S(1)	124.2(2)	C(12')-C(13')-C(14')	120.1(3)
S(3)-C(19)-S(1)	111.08(19)	C(13')-C(14')-C(15')	116.9(3)
C(18')-S(1')-C(19')	97.54(17)	C(13')-C(14')-C(1')	122.2(3)
C(19')-S(3')-C(17')	98.52(17)	C(15')-C(14')-C(1')	119.6(3)
C(14')-C(1')-C(2')	112.0(3)	C(16')-C(15')-C(14')	121.0(3)
C(3')-C(2')-C(1')	113.2(3)	C(15')-C(16')-C(11')	120.3(3)
C(8')-C(3')-C(4')	116.4(3)	C(18')-C(17')-C(4')	126.3(3)
C(8')-C(3')-C(2')	117.7(3)	C(18')-C(17')-S(3')	114.5(3)
C(4')-C(3')-C(2')	124.1(3)	C(4')-C(17')-S(3')	119.1(2)
C(5')-C(4')-C(3')	119.2(3)	C(17')-C(18')-S(1')	117.6(3)
C(5')-C(4')-C(17')	119.4(3)	S(2')-C(19')-S(3')	124.3(2)
C(3')-C(4')-C(17')	121.0(3)	S(2')-C(19')-S(1')	124.0(2)
C(6')-C(5')-C(4')	121.6(3)	S(3')-C(19')-S(1')	111.7(2)

Table S9: Torsion angles [°].

C(14)-C(1)-C(2)-C(3)	-17.2(5)	C(19)-S(3)-C(17)-C(18)	0.5(3)
C(1)-C(2)-C(3)-C(8)	95.1(4)	C(19)-S(3)-C(17)-C(4)	-175.4(3)
C(1)-C(2)-C(3)-C(4)	-68.0(4)	C(4)-C(17)-C(18)-S(1)	175.7(3)
C(8)-C(3)-C(4)-C(5)	-12.7(4)	S(3)-C(17)-C(18)-S(1)	0.1(4)
C(2)-C(3)-C(4)-C(5)	150.7(3)	C(19)-S(1)-C(18)-C(17)	-0.6(3)
C(8)-C(3)-C(4)-C(17)	175.1(3)	C(17)-S(3)-C(19)-S(2)	177.6(2)
C(2)-C(3)-C(4)-C(17)	-21.5(5)	C(17)-S(3)-C(19)-S(1)	-0.8(2)
C(3)-C(4)-C(5)-C(6)	-1.6(5)	C(18)-S(1)-C(19)-S(2)	-177.6(2)
C(17)-C(4)-C(5)-C(6)	171.1(3)	C(18)-S(1)-C(19)-S(3)	0.9(2)
C(4)-C(5)-C(6)-C(7)	15.4(4)	C(14')-C(1')-C(2')-C(3')	23.1(4)
C(4)-C(5)-C(6)-C(9)	-152.7(3)	C(1')-C(2')-C(3')-C(8')	-98.6(4)
C(5)-C(6)-C(7)-C(8)	-14.9(5)	C(1')-C(2')-C(3')-C(4')	65.5(4)
C(9)-C(6)-C(7)-C(8)	153.0(3)	C(8')-C(3')-C(4')-C(5')	15.7(5)
C(6)-C(7)-C(8)-C(3)	0.5(5)	C(2')-C(3')-C(4')-C(5')	-148.7(3)
C(4)-C(3)-C(8)-C(7)	13.3(5)	C(8')-C(3')-C(4')-C(17')	-171.3(3)
C(2)-C(3)-C(8)-C(7)	-151.0(3)	C(2')-C(3')-C(4')-C(17')	24.3(5)
C(5)-C(6)-C(9)-C(10)	87.8(4)	C(3')-C(4')-C(5')-C(6')	-1.3(5)
C(7)-C(6)-C(9)-C(10)	-79.7(4)	C(17')-C(4')-C(5')-C(6')	-174.5(3)
C(6)-C(9)-C(10)-C(11)	-6.0(4)	C(4')-C(5')-C(6')-C(7')	-14.1(5)
C(9)-C(10)-C(11)-C(16)	-80.3(4)	C(4')-C(5')-C(6')-C(9')	154.1(3)
C(9)-C(10)-C(11)-C(12)	86.7(4)	C(5')-C(6')-C(7')-C(8')	14.8(5)
C(16)-C(11)-C(12)-C(13)	14.2(5)	C(9')-C(6')-C(7')-C(8')	-153.3(3)
C(10)-C(11)-C(12)-C(13)	-153.1(3)	C(6')-C(7')-C(8')-C(3')	-0.2(5)
C(11)-C(12)-C(13)-C(14)	-0.2(5)	C(4')-C(3')-C(8')-C(7')	-15.2(5)
C(12)-C(13)-C(14)-C(15)	-13.9(5)	C(2')-C(3')-C(8')-C(7')	150.2(3)
C(12)-C(13)-C(14)-C(1)	152.5(3)	C(7')-C(6')-C(9')-C(10')	73.2(4)
C(2)-C(1)-C(14)-C(15)	96.0(4)	C(5')-C(6')-C(9')-C(10')	-94.5(4)
C(2)-C(1)-C(14)-C(13)	-70.0(4)	C(6')-C(9')-C(10')-C(11')	14.3(5)
C(13)-C(14)-C(15)-C(16)	13.9(5)	C(9')-C(10')-C(11')-C(12')	-94.0(4)
C(1)-C(14)-C(15)-C(16)	-152.7(3)	C(9')-C(10')-C(11')-C(16')	72.4(4)
C(12)-C(11)-C(16)-C(15)	-14.3(5)	C(16')-C(11')-C(12')-C(13')	-15.1(5)
C(10)-C(11)-C(16)-C(15)	153.1(3)	C(10')-C(11')-C(12')-C(13')	151.8(3)
C(14)-C(15)-C(16)-C(11)	0.1(5)	C(11')-C(12')-C(13')-C(14')	0.3(5)
C(5)-C(4)-C(17)-C(18)	-33.6(5)	C(12')-C(13')-C(14')-C(15')	14.7(5)
C(3)-C(4)-C(17)-C(18)	138.8(4)	C(12')-C(13')-C(14')-C(1')	-152.1(3)
C(5)-C(4)-C(17)-S(3)	141.7(3)	C(2')-C(1')-C(14')-C(13')	67.6(4)
C(3)-C(4)-C(17)-S(3)	-45.9(4)	C(2')-C(1')-C(14')-C(15')	-98.8(4)

C(13')-C(14')-C(15')-C(16')	-15.0(5)
C(1')-C(14')-C(15')-C(16')	152.2(3)
C(14')-C(15')-C(16')-C(11')	0.2(5)
C(12')-C(11')-C(16')-C(15')	14.7(5)
C(10')-C(11')-C(16')-C(15')	-152.1(3)
C(5')-C(4')-C(17')-C(18')	-148.0(3)
C(3')-C(4')-C(17')-C(18')	39.0(5)
C(5')-C(4')-C(17')-S(3')	30.6(4)
C(3')-C(4')-C(17')-S(3')	-142.4(3)
C(19')-S(3')-C(17')-C(18')	1.6(3)
C(19')-S(3')-C(17')-C(4')	-177.2(3)
C(4')-C(17')-C(18')-S(1')	179.6(3)
S(3')-C(17')-C(18')-S(1')	0.9(4)
C(19')-S(1')-C(18')-C(17')	-2.9(3)
C(17')-S(3')-C(19')-S(2')	177.7(2)
C(17')-S(3')-C(19')-S(1')	-3.4(2)
C(18')-S(1')-C(19')-S(2')	-177.3(2)
C(18')-S(1')-C(19')-S(3')	3.8(2)
